# Risk factors of ineffective drainage in uncovered self-expandable metal stenting for unresectable malignant hilar biliary strictures

**DOI:** 10.18632/oncotarget.25598

**Published:** 2018-06-15

**Authors:** Koji Takahashi, Toshio Tsuyuguchi, Atsushi Saiga, Takuro Horikoshi, Yoshihiko Ooka, Harutoshi Sugiyama, Masato Nakamura, Junichiro Kumagai, Mutsumi Yamato, Yotaro Iino, Ayako Shingyoji, Hiroshi Ohyama, Shin Yasui, Rintaro Mikata, Yuji Sakai, Naoya Kato

**Affiliations:** ^1^ Department of Gastroenterology, Graduate School of Medicine, Chiba University, Chiba, Japan; ^2^ Department of Radiology, Graduate School of Medicine, Chiba University, Chiba, Japan

**Keywords:** cholangiopancreatography, drainage, endoscopic retrograde, risk factor, stent

## Abstract

**Aim:**

In this study, we assessed the factors contributing to ineffective drainage in the initial transpapillary uncovered self-expandable metal stent (USEMS) placements in patients with unresectable malignant hilar biliary strictures (UMHBSs) (Bismuth type II or higher).

**Methods:**

This was a retrospective, single-center study. A total of 97 patients with UMHBSs who underwent technically successful initial USEMS placements using endoscopic retrograde cholangiopancreatography (ERCP) were classified into the effective drainage group (n = 73) or the ineffective drainage group (n = 24). We then compared group characteristics, clinical outcomes, and drained liver volumes. Drained liver volume was measured by using computed tomography volumetry. The definition of effective biliary drainage was a 50% decrease in the serum total bilirubin level or normalization of the level within 14 days of stent placement.

**Results:**

Univariate analysis showed that ineffective drainage was associated with the pre-ERCP serum total bilirubin level (*P* = 0.0075), pre-ERCP serum albumin level (*P* = 0.042), comorbid liver cirrhosis (*P* = 0.010), drained liver volume (*P* = 0.0010), and single stenting (*P* = 0.022). Multivariate analysis identified comorbid liver cirrhosis (adjusted odds ratio [OR], 5.79; 95% confidence interval [CI], 1.30–25.85; *P* = 0.022) and drained liver volume < 50% (adjusted OR, 5.50; 95% CI, 1.50–20.25; *P* = 0.010) as independent risk factors of ineffective drainage.

**Conclusion:**

Comorbid liver cirrhosis and a drained liver volume < 50% contributed significantly to ineffective drainage in the initial transpapillary USEMS placements for UMHBSs.

## INTRODUCTION

A malignant hilar biliary stricture (MHBS) is caused by cholangiocarcinoma, gallbladder carcinoma, metastatic liver tumors, or hilar lymph node metastases from various cancers. MHBSs induce high serum bilirubin concentrations due to cholestasis (obstructive jaundice). Obstructive jaundice affects the biliary tree, and the hepatic cell and liver functions. The loss of bile in the gut disrupts the intestinal mucosal barrier, which increases the absorption of endotoxin from the intestinal tract. The resulting endotoxemia causes inflammatory cytokinesis and induces a systemic inflammatory response syndrome, which may lead to a multiple organ dysfunction syndrome [1].

**Figure 1:**
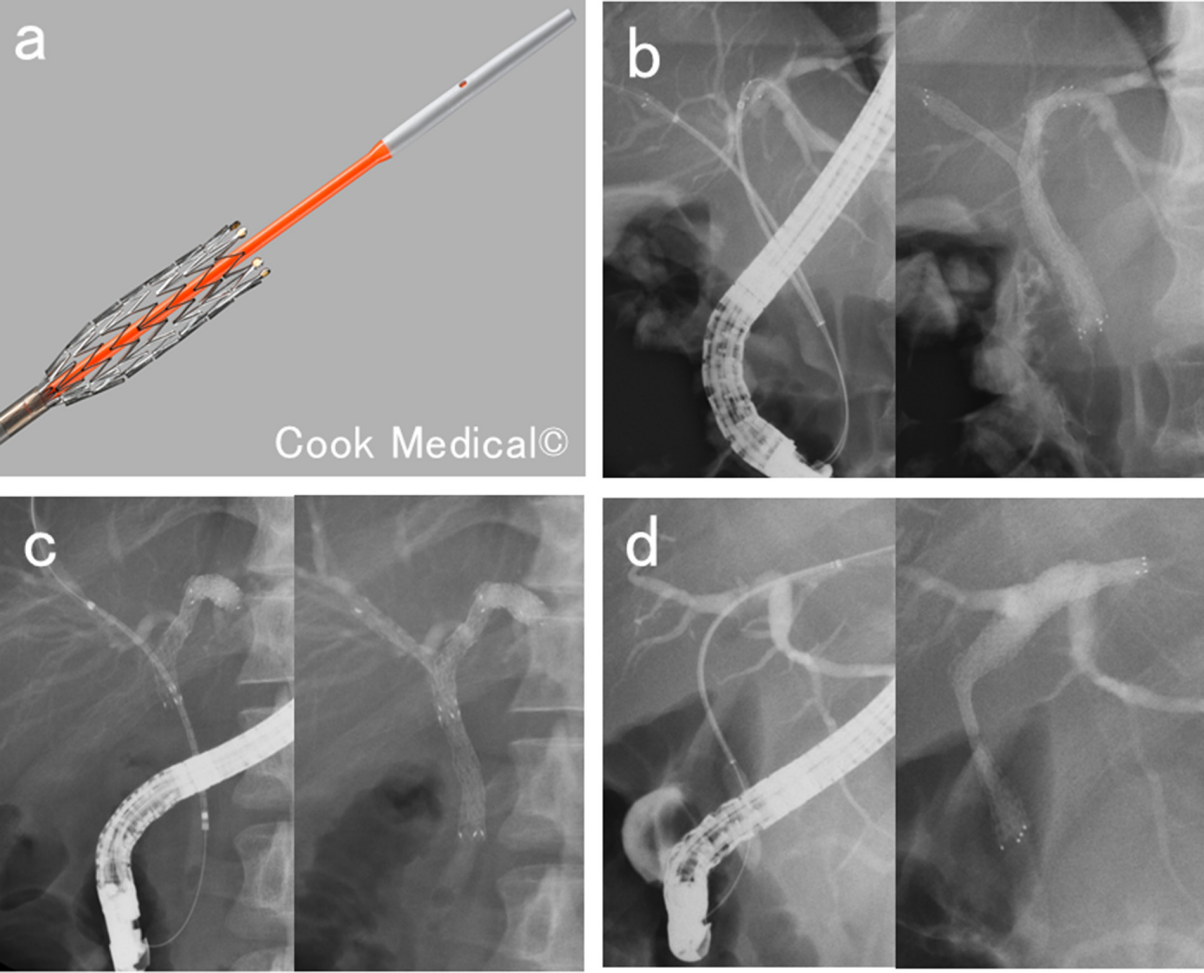
Methods of biliary metal stenting for malignant hilar biliary strictures **(a)** Uncovered self-expandable metal stent and its delivery system. **(b)** Side-by-side placement. Two metal stents inserted parallel to each other into different branches of the hepatic bile duct. **(c)** Partial stent-in-stent placement. A second metal stent inserted into a different branch of the hepatic bile duct system through the mesh of the first metal stent. **(d)** Single stenting. Metal stent inserted into one branch of the hepatic bile duct.

Effective biliary drainage is essential to improve the quality of life of patients with MHBS. Plastic and metallic stents (MSs) are available for biliary drainage in patients with unresectable MHBSs (UMHBSs), and studies have shown that MSs are superior in terms of their patency period and cost effectiveness [2, 3]. Thus, uncovered self-expandable MSs (USEMSs) are now mainly used for drainage in patients with UMHBSs. Metal stenting methods are diverse and include side-by-side placement (SBS), partial stent-in-stent placement (PSIS), and single stenting (Figure 1). However, there are no unified guidelines with regard to the drained liver volume and the stenting method involving a MS for a UMHBS [4]. Furthermore, the factors contributing to ineffective drainage in the initial transpapillary USEMS placements in patients with UMHBSs are unclear. In the TOKYO Criteria 2014, functional success, which indicates effective biliary drainage, was defined as a 50% decrease in the serum total bilirubin level or normalization of the serum total bilirubin level within 14 days of stent placement [5]. This study assessed factors contributing to ineffective drainage in the initial transpapillary USEMS placements in patients with UMHBSs (Bismuth type II or higher).

## RESULTS

**Figure 2:**
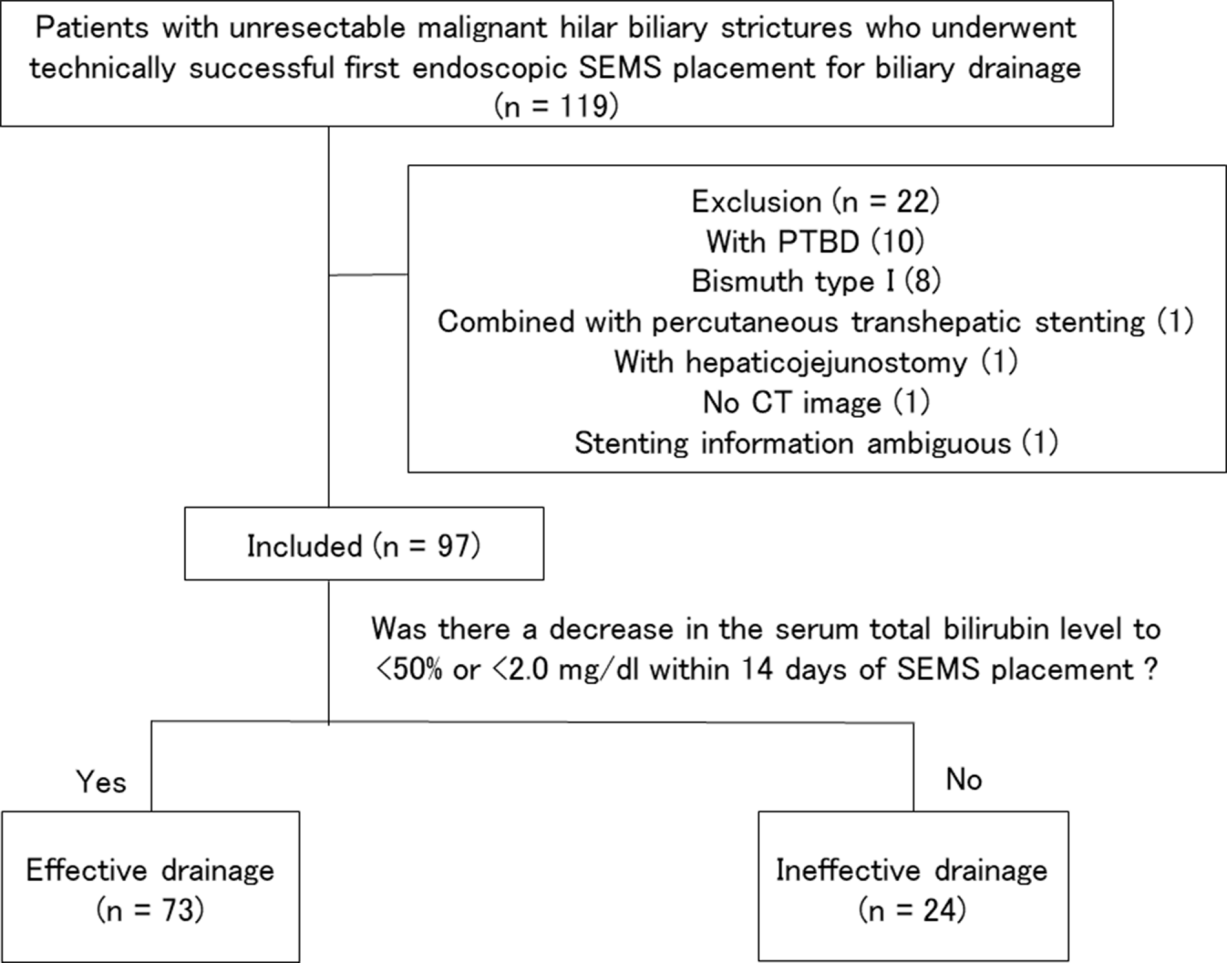
Diagram of case selection flow

A total of 97 patients with UMHBSs who underwent technically successful first-time endoscopic SEMS placement for biliary drainage at the Chiba University Hospital between July 2005 and September 2017 contributed data for this analysis (Figure 2). The patients’ characteristics and treatment outcomes are shown in Table 1. There were 67 men and 30 women (median [interquartile range, IQR] age, 69 [63–78] years). Of the 97 patients, 34 underwent SBS, 18 underwent PSIS, and 45 underwent single stenting. For SBS and PSIS, no cases underwent stent placements in three or more branches of the bile duct in a single procedure. All patients had undergone endoscopic sphincterotomy before the SEMS placements. No patients had marked thrombocytopenia, and no patients had dialysis requirements.

**Table 1:** Clinical features of the 97 patients

Characteristics and clinical outcomes	
Age, median (IQR)	69 (63–78)
Gender, male, n (%)	67 (69%)
Performance status, median (IQR)	1 (1–3)
Indications, n (%)	
Cholangiocarcinoma	54 (56%)
Gallbladder carcinoma	17 (18%)
Hepatocellular carcinoma	14 (14%)
Metastatic liver tumor	10 (10%)
Hilar lymph nodes metastasis	2 (2%)
Method of previous biliary drainage, n (%)	
No history of previous biliary drainage	23 (24%)
Plastic stent	37 (38%)
ENBD	36 (37%)
PTBD	1(1%)
Pre-ERCP serum total bilirubin level, mg/dl, median (IQR)	4.8 (1.7–9.8)
Pre-ERCP serum albumin level, mg/dl, median (IQR)	2.9 (2.6–3.3)
Comorbid cholangitis, n (%)	18 (19%)
Comorbid liver cirrhosis, n (%)	16 (16%)
Bismuth classification type, n (%)	
Type II	12 (12%)
Type III	20 (21%)
Type IV	65 (67%)
Stenting method, n (%)	
Side-by-side placement	34 (35%)
Partial stent-in-stent placement	18 (19%)
Single stenting	45 (46%)
Stent placement above the papilla, n (%)	90 (93%)
Drained liver volume, %, median (IQR)	53.5 (42.3–61.8)
Effective drainage, n (%)	73 (75%)
Complication, n (%)	10 (10%)

Abbreviations: IQR, interquartile range; ENBD, endoscopic retrograde biliary drainage; PTBD, percutaneous transhepatic biliary drainage; ERCP, endoscopic retrograde cholangiopancreatography

Of the 97 eligible patients, data from 73 were included in the effective drainage group, and data from 24 were included in the ineffective drainage group (Table 2). Univariate analysis identified the significant factors contributing to ineffective drainage as the pre- endoscopic retrograde cholangiopancreatography (ERCP) serum total bilirubin level (*P* = 0.0075), pre-ERCP serum albumin level (*P* = 0.042), comorbid liver cirrhosis (*P* = 0.010), drained liver volume (*P* = 0.0010), and single stenting (*P* = 0.022). The types of indications, methods of previous biliary drainage, and Bismuth classification types were not significant contributing factors.

**Table 2:** Univariate analysis for risk factors of ineffective biliary drainage

	Effective drainage	Ineffective drainage	*P*-value
	n = 73	n = 24	
Age, median (IQR)	71 (64–79)	67 (61–71)	0.056
Gender, male, n (%)	49 (67%)	18 (75%)	0.47
Performance status, median (IQR)	1 (1–3)	1 (1–2)	0.27
Cholangiocarcinoma, n (%)	44 (60%)	10 (42%)	0.11
Gallbladder carcinoma, n (%)	12 (16%)	5 (21%)	0.62
Hepatocellular carcinoma, n (%)	10 (14%)	4 (17%)	0.72
Metastatic liver tumor, n (%)	6 (8%)	4 (17%)	0.24
Hilar lymph nodes metastasis, n (%)	1 (1%)	1 (4%)	0.40
Method of previous biliary drainage, n (%)			
No history of previous biliary drainage, n (%)	15 (21%)	8 (33%)	0.20
Plastic stent	31 (42%)	6 (25%)	0.13
ENBD	27 (37%)	9 (38%)	0.96
PTBD	0	1 (4%)	0.080
Pre-ERCP serum total bilirubin level, mg/dl, median (IQR)	3.9 (1.5–7.7)	8.2 (4.6–13.9)	0.0075
Pre-ERCP serum albumin level, mg/dl, median (IQR)	2.9 (2.7–3.3)	2.7 (2.1–3.2)	0.042
Comorbid cholangitis, n (%)	11 (15%)	7 (29%)	0.12
Comorbid liver cirrhosis, n (%)	8 (11%)	8 (33%)	0.010
Bismuth classification type, n (%)			
Type II	10 (14%)	2 (8%)	0.49
Type III	14 (19%)	6 (25%)	0.54
Type IV	49 (67%)	16 (67%)	0.97
Drained liver volume, %, median (IQR)	56 (49–64)	40 (34–51)	0.0010
Side-by-side placement, n (%)	29 (40%)	5 (21%)	0.092
Partial stent-in-stent placement, n (%)	15 (21%)	3 (13%)	0.38
Single stenting, n (%)	29 (40%)	16 (67%)	0.022
Stent placement above the papilla, n (%)	67 (92%)	23 (96%)	0.51

Abbreviations: IQR, interquartile range; ENBD, endoscopic retrograde biliary drainage; PTBD, percutaneous transhepatic biliary drainage; ERCP, endoscopic retrograde cholangiopancreatography.

**Figure 3:**
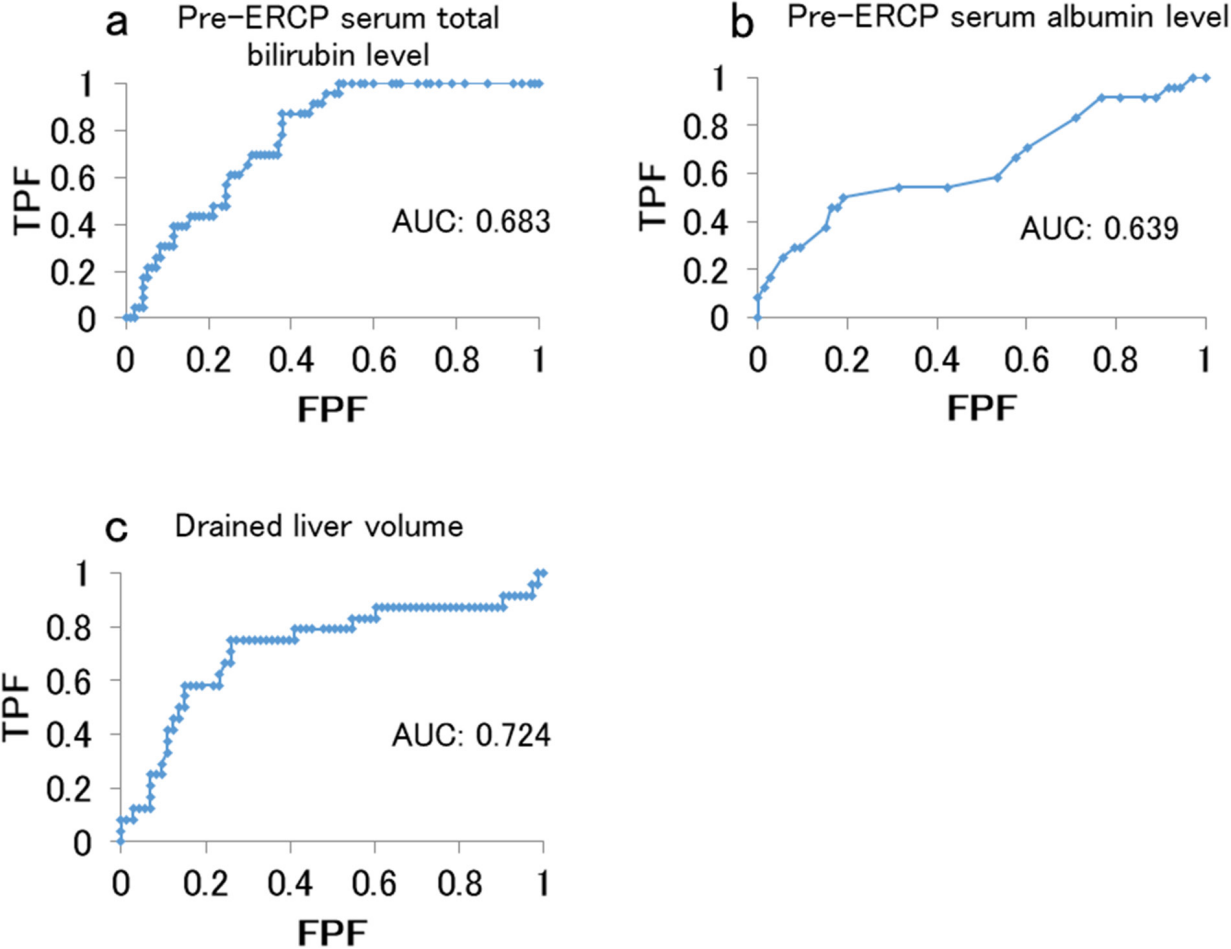
Area under the receiver operating characteristic curve for predicting effective drainage **(a)** Pre-stenting serum total bilirubin level. **(b)** Pre-stenting serum albumin level. **(c)** Drained liver volume. Abbreviations: FPF, false positive fraction; TPF, true positive fraction.

The areas under the receiver operating characteristic (ROC) curve for the pre-ERCP serum total bilirubin level, pre-ERCP serum albumin level, and drained liver volume were 0.683, 0.639, and 0.724, respectively. We calculated cutoff values for the pre-ERCP serum total bilirubin level (4.8 mg/dl; sensitivity, 75%; specificity, 68%), pre-ERCP serum albumin level (2.6 mg/dl; sensitivity, 50%; specificity, 81%), and drained liver volume were (50%; sensitivity, 75%; specificity, 74%) (Figure 3). We classified the pre-ERCP serum total bilirubin level, pre-ERCP serum albumin level, and drained liver volume according to the cutoff values (Table 3), and then performed a multivariate analysis.

**Table 3:** Rate of ineffective drainage when divided into two groups according to the cut-off value

	Effective drainage	Ineffective drainage	*P*-value
	n = 73	n = 24	
Pre-ERCP serum total bilirubin level ≥4.8 mg/dl, n (%)	31 (42%)	18 (75%)	0.0057
Pre-ERCP serum albumin level ≤2.6 mg/dl, n (%)	15 (21%)	12 (50%)	0.0052
Drained liver volume <50%, n (%)	19 (26%)	17 (71%)	0.0001

Abbreviations: ERCP, endoscopic retrograde cholangiopancreatography

The multivariate analysis used the five items that had shown significance in the univariate analysis (Table 4). Our multivariate analysis identified comorbid liver cirrhosis (adjusted odds ratio [OR], 5.79; 95% confidence interval [CI], 1.30–25.85; *P* = 0.022) and drained liver volume < 50% (adjusted OR, 5.50; 95% CI, 1.50–20.25; *P* = 0.010) as independent risk factors of ineffective drainage.

**Table 4:** Multivariate analysis for the risk factors of ineffective biliary drainage

	Adjusted odds ratio	95% confidence interval		*P*-value
		Lower	Upper	
Pre-ERCP serum total bilirubin level ≥4.8 mg/dl	3.45	0.96	12.42	0.058
Pre-ERCP serum albumin level ≤2.6 mg/dl	3.15	0.88	11.29	0.077
Single stenting	1.42	0.36	5.55	0.620
Comorbid of liver cirrhosis	5.79	1.30	25.85	0.022
Drained liver volume <50%	5.50	1.50	20.25	0.010

Abbreviations: ERCP, endoscopic retrograde cholangiopancreatography

Complications occurred in 10 patients (10%). Five patients had cholangitis, two had pancreatitis, and one each had hepatic abscess, pneumonia, and heart failure. In the patient with a hepatic abscess, the abscess formed in a non-drained area, and additional stenting was required. The other complications in patients were quickly relieved, and no deaths occurred in association with the SEMS placement.

## DISCUSSION

Our study results identified comorbid liver cirrhosis and drained liver volume < 50% as significant risk factors of ineffective drainage.

Reports have mentioned that single stenting is sufficient for reducing the bilirubin level of patients with MHBSs [6, 7]. A prospective study on 35 patients showed that unilateral metal stenting using magnetic resonance cholangiopancreatography or computed tomography (CT) to selectively target drainage, provided safe and effective palliation in most patients with MHBSs [6]. Another prospective study on 61 patients showed that unilateral MS placement was safe and feasible and achieved adequate drainage in most patients with unresectable hilar cholangiocarcinoma [7]. In recent years, many studies have reported on the efficacy of multiple stenting [8–13]. However, there are few reports on factors contributing to ineffective drainage, and these factors are not clear. Drained liver volume is considered to be important for effective drainage, and drained liver volume has been assessed using CT volumetry [14, 15]. Vennie et al. reported that drainage > 50% of the variable liver volume is an important predictor of drainage and signals effective palliation in patients with MHBSs [14]. Takahashi et al. reported that a liver volume drainage ≥ 33% in patients with preserved liver function correlates with effective biliary drainage in cases of UMHBSs [15]. Both these studies employed various biliary drainage methods. However, our study focused on only the initial USEMS placements in patients with UMHBSs. To our knowledge, this is the first study to investigate the factors contributing to ineffective drainage after the initial metal stenting for UMHBSs.

Our multivariate analysis showed that comorbid liver cirrhosis and drained liver volume < 50% were independent significant factors contributing to ineffective drainage. Based on that, a stenting method draining ≥ 50% of the liver volume should be planned for the first transpapillary USEMS placement in patients with UMHBSs (Bismuth type II or higher). In cases with Bismuth type II or III, this is achievable with single stenting, but in Bismuth type IV cases, multiple stenting is necessary to drain ≥50% of the liver volume, and multiple stenting should be attempted. It should also be noted that effective drainage is more difficult in patients with liver cirrhosis than in patients with a normal liver. A larger drainage area seems to be required in patients with impaired liver function than in patients with preserved liver function [15]. In this study, we took the presence of liver cirrhosis into consideration, and impaired liver function or preserved liver function was not classified. The required drained liver volume for effective drainage may change in relation to the liver functionality, and for patients with cirrhosis, a drained liver volume > 50% might be necessary. The number of patients with liver cirrhosis was small in our study, and a larger study is required to assess the conditions necessary for effective drainage in patients with cirrhosis.

We are aware of the limitations in our study. First, this is a retrospective study at a single-center and the number of patients whose data we analyzed was limited to 97. Second, our study population targeting patients with different types of diseases was heterogeneous. Finally, our CT volumetry may not be the most accurate test for evaluating drained liver volume, particularly in patients with high stenosis, and the assessment method should be improved for future studies.

In conclusion, the significant factors contributing to ineffective drainage after an initial transpapillary USEMS placement for UMHBSs are comorbid liver cirrhosis and drained liver volume < 50%. The strategy for the first transpapillary USEMS placement in patients with UMHBSs (Bismuth type II or higher) should involve stenting for draining ≥ 50% of the liver volume to achieve effective drainage. A prospective study is needed to validate the results of our study.

## PATIENTS AND METHODS

### Study design

This was a retrospective, single-center study. We reviewed the medical records of patients and compared the characteristics and clinical outcomes between those with effective drainage (effective drainage group) and those without effective drainage (ineffective drainage group) after USEMS placement for a UMHBS. Additionally, we calculated the estimated drained liver volume using CT volumetry. Moreover, we compared various factors between the two groups. Significant factors contributing to ineffective drainage were identified in a multivariate analysis. The ethics committee of the Chiba University Hospital approved this study.

### Patients

Patients with UMHBSs who underwent technically successful first-time endoscopic SEMS placement for biliary drainage at the Chiba University Hospital between July 2005 and September 2017 were considered for inclusion. We identified a total of 119 consecutive patients through retrospective analysis of the prospectively recorded endoscopic database in our hospital. Technical success was defined as SEMS placement with sufficient coverage of the bile duct stricture. The exclusion criteria were as follows: percutaneous transhepatic biliary drainage was continued even after metal stenting, Bismuth type I condition, percutaneous transhepatic metal stenting, biliary tract reconstruction, no abdominal CT scan within 14 days before stenting, and ambiguous stenting information.

### Techniques

Transpapillary SEMS placements for biliary drainage were performed via therapeutic duodenoscopy (JF 260 V or TJF 260 V; Olympus, Tokyo, Japan) using the ERCP technique. The SEMSs included Zilver635 biliary stent (Cook Japan, Tokyo, Japan), X-Suit NIR (Olympus, Tokyo, Japan), WallFlex (Boston Scientific Japan, Tokyo, Japan), Niti-S D-type (Century Medical, Tokyo, Japan), BILERUSH (PIOLAX, Yokohama, Japan), and JOSTENT (Zeon Medical, Tokyo, Japan). In cases of SBS and PSIS, which require the stents to be place into two branches of the bile duct, the stenting was completed with a single ERCP in all relevant cases. In this study, the SBS and PSIS were considered as multiple stenting procedures. On the other hand, stenting in a single branch of the bile duct was considered as single stenting.

### Methods

For this study, patients satisfying the functional success criteria of the TOKYO Criteria 2014 without additional interventions were included in the effective drainage group, and the remaining patients were included in the ineffective drainage group. We defined functional success as a decrease in the serum total bilirubin level to < 50% or < 2.0 mg/dl within 14 days of SEMS placement without additional biliary treatments. We compared the clinical characteristics, stenting methods, clinical outcomes, and estimated drained liver volumes between the groups. We analyzed the following variables in the effective and ineffective drainage groups: age, gender, Eastern Cooperative Oncology Group performance status, types of indications, Bismuth types [16], stenting methods, complications, pre-ERCP serum total bilirubin levels, pre-ERCP serum albumin levels, comorbid rates of cholangitis, comorbid rates of liver cirrhosis, and estimated drained liver volumes. The Bismuth types were based on the retrograde cholangiography and CT findings comprehensively. We evaluated the complication events according to the TOKYO Criteria 2014. Liver cirrhosis was judged by two gastroenterologists. Patients with medical history and laboratory data suggestive of cirrhosis and findings characteristic of liver cirrhosis on abdominal ultrasound, CT, or magnetic resonance imaging were considered to have liver cirrhosis.

**Figure 4:**
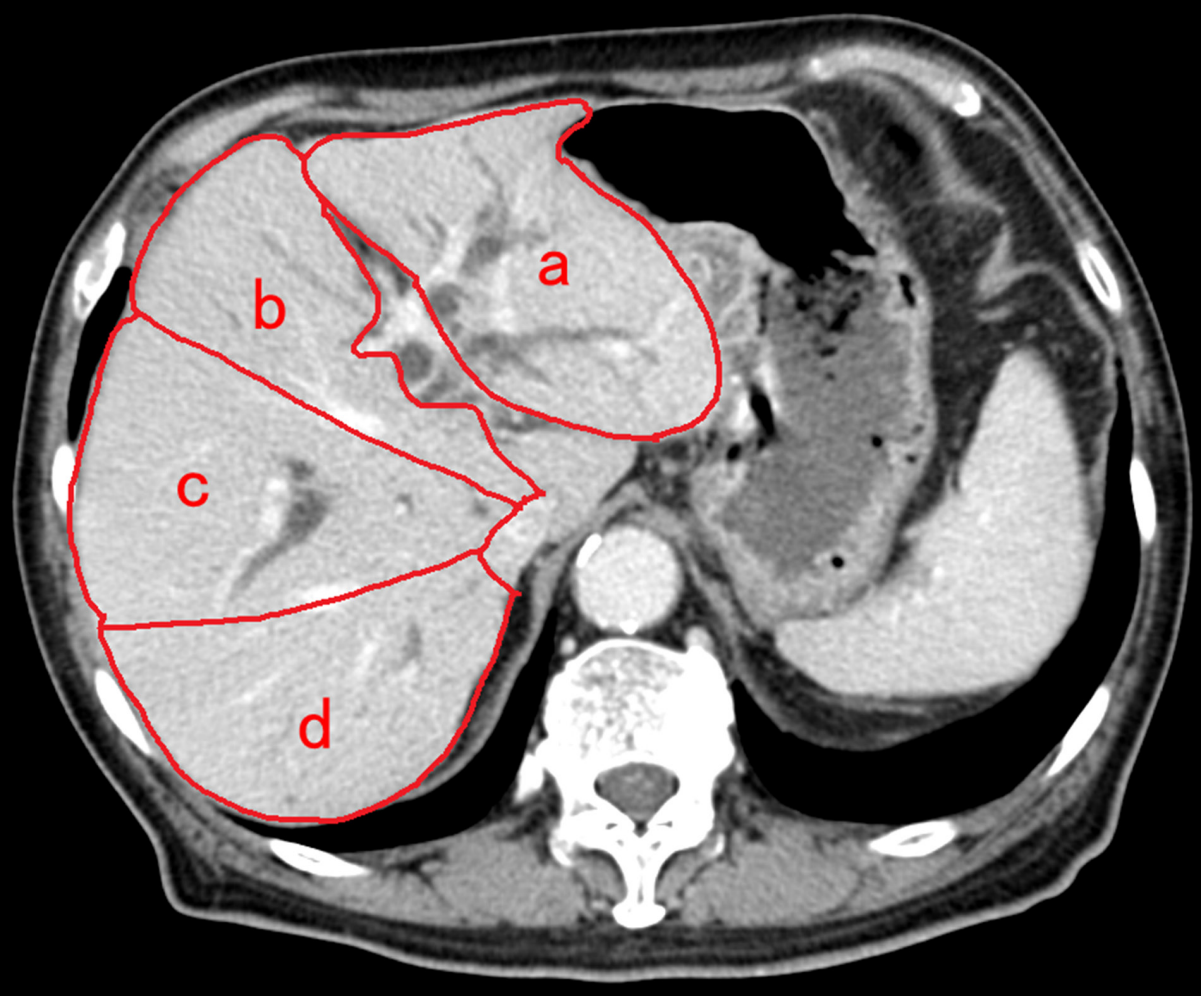
Method of identification of each liver section and computed tomography volumetry Four sectors of liver were defined based on the distribution of portal vein branches, excluding the caudate lobe. The areas of the left lateral sector **(a)**, left medial sector **(b)**, right anterior sector **(c)**, and right posterior sector **(d)** were measured in computed tomography (CT) images. Additionally, the area of each sector was measured by tracing CT images, and then summing up the area of the CT slices comprising that sector.

For measurement of estimated drained liver volume using CT volumetry, we referred to previous reports [14, 15] and consultation was performed with the radiological department and gastroenterological department in Chiba University Hospital, and then, the assessment method was decided. According to the distribution of portal vein branches, four sectors of the liver were defined, excluding the caudate lobe. The sectors were the left lateral sector, left medial sector, right anterior sector, and right posterior sector. The areas of these sectors were measured in CT images (Figure 4). We manually traced the area of each sector using axial CT images with a 5-mm slice thickness, including the tumor component, to calculate the volume of each sector (summed area of the slices comprising the sector). Then, we calculated the drained liver volume, which did not include the tumor component, on the basis of the stent position, the non-tumor volume of each liver sector, and the type of bile duct stricture, according to the Bismuth classification. Additionally, we calculated the ratios of the drained liver volume to the total live volume.

With regard to the effectiveness of previous drainage, some cases changed from previous drainage to USEMSs within a few days, and thus, it was difficult to evaluate the effectiveness of previous drainage.

### Statistical analysis

We performed univariate analysis for comparisons between the effective and ineffective drainage groups. Pearson's chi-squared test was used to assess categorized data, while the Mann–Whitney *U* test was used to assess quantitative data. For multivariate analysis, we performed binomial logistic regression analysis on items that showed significance in the univariate analysis. For quantitative data, categorization was performed using cutoff values calculated by determining the smallest distance between the receiver operating characteristic (ROC) curve and the upper left corner of the graph, and multivariate analysis was performed using that categorized data. Data are presented as median (interquartile range [IQR]) or number (%). A *P*-value < 0.05 was considered statistically significant. All statistical analyses were performed using BellCurve for Excel (Social Survey Research Information Co., Ltd., Tokyo, Japan).

Endoscopic operation: Koji Takahashi, Toshio Tsuyuguchi, Harutoshi Sugiyama, Masato Nakamura, Junichiro Kumagai, Mutsumi Yamato, Yotaro Iino, Hiroshi Ohyama, Shin Yasui, Rintaro Mikata, and Yuji Sakai.

Image analysis: Koji Takahashi, Toshio Tsuyuguchi, Atsushi Saiga, and Takuro Horikoshi.

Postoperative management and patient follow-up: Koji Takahashi, Toshio Tsuyuguchi, Harutoshi Sugiyama, Masato Nakamura, Junichiro Kumagai, Mutsumi Yamato, Yotaro Iino, Ayako Shingyoji, Hiroshi Ohyama, Shin Yasui, Rintaro Mikata.

Drafting of the manuscript: Koji Takahashi, Toshio Tsuyuguchi, and Yoshihiko Ooka.

## Abbreviations

USEMS: uncovered self-expandable metal stent
UMHBS: unresectable malignant hilar biliary stricture
ERCP: endoscopic retrograde cholangiopancreatography
MHBS: malignant hilar biliary stricture
MS: metal stent
SBS: side-by-side placement
PSIS: partial stent-in-stent placement
CT: computed tomography
ROC: receiver operating characteristic
IQR: interquartile range.
